# MRI Radiomics-Based Nomogram Integrating Skeletal Muscle Assessment for Predicting Pathologic Complete Response to Neoadjuvant Therapy in Triple-Negative Breast Cancer

**DOI:** 10.3390/diagnostics16213679

**Published:** 2026-11-06

**Authors:** Han Song Mun, Ga Eun Park, Bong Joo Kang, Kabsoo Shin, Soo Youn Bae, Jinwoo Hwang, Misun Park, Sung Hun Kim

**Affiliations:** 1 Department of Radiology, Seoul St. Mary’s Hospital, College of Medicine, The Catholic University of Korea, Seoul 06951, Republic of Korea; im_hsm@catholic.ac.kr (H.S.M.); hoonhoony@naver.com (G.E.P.); lionmain@gmail.com (B.J.K.); 2 Division of Medical Oncology, Department of Internal Medicine, Seoul St. Mary’s Hospital, College of Medicine, The Catholic University of Korea, Seoul 06951, Republic of Korea; kabsoo.shin@catholic.ac.kr; 3 Department of Surgery, Seoul St. Mary’s Hospital, College of Medicine, The Catholic University of Korea, Seoul 06951, Republic of Korea; baessu@gmail.com; 4 Philips Healthcare, Seoul 04637, Republic of Korea; j.w.hwang@philips.com; 5 Department of Biostatistics, Clinical Research Coordinating Center, The Catholic University of Korea, Seoul 06591, Republic of Korea; misunpark@cmcnu.or.kr

**Keywords:** breast neoplasms, neoadjuvant therapy, radiomics, nomograms, treatment outcome

## Abstract

**Background/Objectives**: We aim to develop and internally evaluate a magnetic resonance imaging (MRI) radiomics-based nomogram integrating clinicopathologic and skeletal muscle measures for predicting pathologic complete response (pCR) in triple-negative breast cancer (TNBC). **Methods:** This retrospective study included 174 patients with stage II–III TNBC receiving neoadjuvant chemoimmunotherapy (*n* = 94) or chemotherapy alone (*n* = 80), followed by surgery. Pretreatment MRI features underwent redundancy reduction and least absolute shrinkage and selection operator (LASSO) selection. Six candidate models combined radiomic, clinicopathologic, and skeletal muscle predictors. Models were fitted using Firth penalized logistic regression. Internal validation used 1000 bootstrap resamples, repeating supervised feature selection, predictor selection, and final-model selection while keeping the 325 radiomic candidates fixed. Area under the curve (AUC), calibration, prediction error, and selection stability were evaluated. **Results**: Eighty-five patients (48.9%) achieved pCR. Seven radiomic features constituted the score. Corrected AUCs were 0.659 for the clinicopathologic model, 0.622 after adding radiomics, and 0.629 (95% CI: 0.500–0.701) for the fully integrated model (apparent AUC, 0.793). Its corrected Brier score (0.309) exceeded the constant-prevalence benchmark (approximately 0.250). Skeletal muscle density was not independently associated with pCR (*p* = 0.130) and did not significantly improve AUC over the radiomics-plus-clinicopathologic model (ΔAUC = 0.009, *p* = 0.428). The fully integrated model was selected in 50.3% of resamples, while 99.9% of selected models contained radiomic information. **Conclusions**: The radiomic score showed exploratory associations with pCR, but optimism-corrected performance and model stability were limited. Skeletal muscle density did not significantly improve discrimination. External validation is required before clinical application.

## 1. Introduction

Triple-negative breast cancer (TNBC) constitutes approximately 15% of all breast cancer cases [1] and is recognized for its aggressive behavior, being associated with an elevated risk of early recurrence and distant metastasis compared to other molecular subtypes [2]. For stage II–III TNBC, neoadjuvant systemic therapy is the standard of care, providing tumor downstaging as well as prognostic stratification [3,4,5]. Pathologic complete response (pCR) represents a widely accepted surrogate endpoint in TNBC, with reported predictors frequently including high Ki-67 expression, programmed death-ligand 1 (PD-L1) expression, and a high density of tumor-infiltrating lymphocytes—especially CD8+ T cells—alongside related immune gene signatures [6].

Following the outcomes of the KEYNOTE-522 trial, the present standard of care for stage II–III TNBC is neoadjuvant chemoimmunotherapy (NACI), comprising chemotherapy combined with immune checkpoint inhibition, which results in pCR rates of approximately 65% [7,8]. In contrast to the metastatic setting [9], patients with early-stage TNBC are eligible for pembrolizumab irrespective of PD-L1 status, with both pCR and event-free survival improvements seen in PD-L1–positive and PD-L1–negative cohorts. Despite this, existing biomarkers, including PD-L1, do not offer reliable response prediction, underscoring the demand for superior predictive models.

Artificial intelligence and quantitative imaging are increasingly extending breast imaging beyond visual interpretation toward computer-aided detection, lesion characterization, and outcome prediction. Advanced magnetic resonance imaging (MRI) modalities, including dynamic contrast-enhanced (DCE) MRI, diffusion-weighted imaging (DWI), and radiomics, have demonstrated potential for forecasting pCR in breast cancer [10,11]. Data from the ACRIN 6657 trial [12] established that MRI provided more accurate evaluation of tumor size after NAC than clinical examination or mammography. Kwak et al. [13] reported moderate predictive ability, while an MRI radiomics nomogram combining imaging and clinicopathologic characteristics achieved an AUC of 0.86 in an external validation cohort [14]. Beyond conventional breast imaging, computer-aided approaches using hyperspectral imaging and other emerging optical techniques further illustrate the broader evolution of AI-assisted breast cancer detection and tissue characterization [15,16].

In addition to tumor features, patient-related factors, such as skeletal muscle measures, have also been associated with breast cancer prognosis [17,18,19,20,21,22]. Sarcopenia is a progressive, generalized skeletal muscle disorder involving loss of muscle strength and muscle quantity or quality [23]. Although sarcopenia has consistently correlated with increased chemotherapy-related toxicity and reduced survival [24], its capacity to predict pCR after NAC has produced inconsistent findings [25,26,27]. Recently, our work demonstrated that skeletal muscle density (SMD), as opposed to muscle mass, was independently linked to elevated pCR rates and better survival in TNBC patients receiving NACI, whereas no such link was observed among those who received NAC alone [17]. These observations suggest skeletal muscle quality may serve as a valuable predictor in the context of immunotherapy. However, previous radiomics-based nomograms for pCR prediction have largely relied on tumor-derived imaging features and clinicopathologic characteristics, without incorporating host-related factors. To date, skeletal muscle parameters have not been integrated into radiomics-based predictive models for TNBC.

In this investigation, we developed and evaluated several models that integrate MRI radiomics, clinicopathologic and treatment-related variables, and skeletal muscle metrics. Accordingly, this study aimed to develop and internally evaluate a nomogram based on the selected model for estimating pCR in patients with TNBC.

## 2. Materials and Methods

### 2.1. Study Population

**Figure 1:**
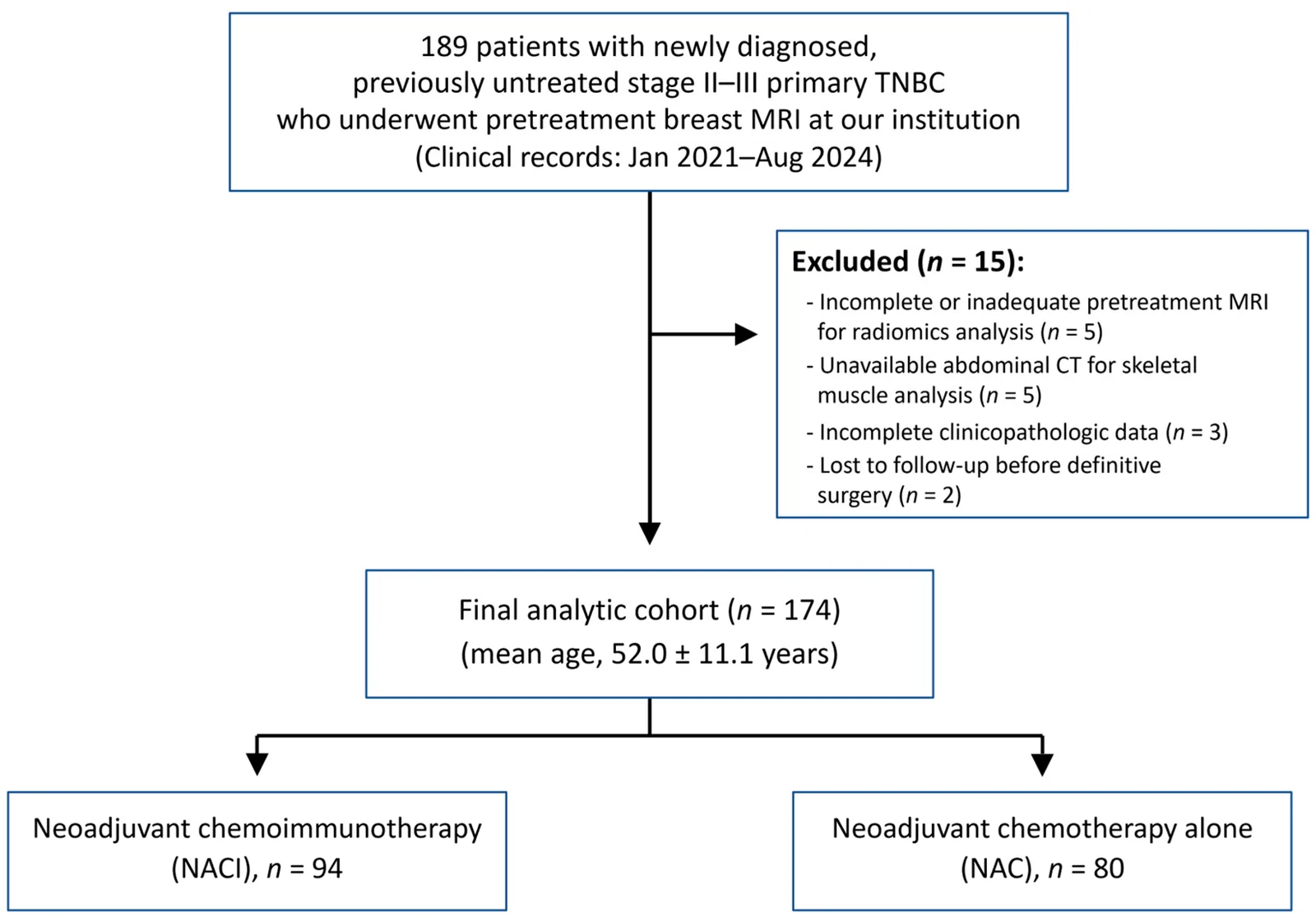
Study flowchart of patient selection. TNBC = triple-negative breast cancer; MRI = magnetic resonance imaging; CT = computed tomography.

We retrospectively identified 189 consecutive patients with TNBC who underwent pretreatment breast MRI and received neoadjuvant therapy at our institution, with definitive surgery planned at our institution. Clinical records from January 2021 through August 2024 were reviewed, including subsequent surgical and pathological outcomes. The eligibility criteria were: (1) newly diagnosed primary TNBC confirmed on pretreatment core needle biopsy; (2) clinical stage II–III disease; (3) no prior locoregional or systemic treatment for breast cancer; and (4) treatment with a standard neoadjuvant regimen of paclitaxel/carboplatin followed by doxorubicin/cyclophosphamide, administered either with pembrolizumab (NACI group) or without pembrolizumab (NAC group). Of these 189 patients, 15 were excluded because of an incomplete pretreatment MRI examination or inadequate image quality for radiomics analysis (*n* = 5), unavailable abdominal CT images required for skeletal muscle analysis (*n* = 5), incomplete clinicopathologic data (*n* = 3), or loss to follow-up before definitive surgery (*n* = 2). The final analytic cohort comprised 174 patients who underwent definitive surgery and had pretreatment breast MRI suitable for radiomics analysis, including 94 patients in the NACI group and 80 in the NAC group (Figure 1).

The present cohort partially overlapped with that of our previous study [17], which included 144 patients treated between March 2021 and March 2024. Specifically, 132 of the 174 patients (75.9%) in the present cohort had also been included in the previous study (NACI, *n* = 94; NAC, *n* = 38). Of the 144 patients in the previous cohort, 12 were not included in the present study, whereas 42 patients in the present cohort had not been included in the previous study. The present cohort was identified according to the eligibility criteria and study period of the current study, including the requirement for pretreatment breast MRI performed at our institution. The present study also addresses a distinct research question by incorporating pretreatment MRI radiomics, clinicopathologic variables, and skeletal muscle measures into a multivariable model for predicting pCR.

This study was approved by the Institutional Review Board of Seoul St. Mary’s Hospital, The Catholic University of Korea (approval No. KC24RISI0533; 13 August 2024). The study was conducted in accordance with the principles of the Declaration of Helsinki. ChatGPT (OpenAI, GPT-5.5) was used to assist with English language editing, including improvements to grammar and wording. It was not used for data collection, statistical analysis, interpretation of the results, or generation of scientific conclusions. All AI-assisted text was reviewed and edited by the authors, who take full responsibility for the manuscript.

### 2.2. Clinicopathologic Characteristics

Clinical data were obtained from electronic health records and included age (years), menopausal status (premenopausal or postmenopausal), family history of breast cancer (no or yes), BRCA1/2 mutation status (absent or present), clinical T category (cT1–4), clinical N category (cN0–3), and overall clinical stage (II or III).

Tumor type (invasive ductal or other), tumor grade (I–II or III), Ki-67 labeling index, and PD-L1 status were determined from initial biopsy pathology prior to neoadjuvant therapy. Ki-67 was dichotomized as high (≥20%) or low (<20%) as defined by the 2013 St. Gallen consensus [28]. PD-L1 expression was evaluated by immunohistochemistry using the 22C3 pharmDx assay (Dako, Santa Clara, CA, USA), and the combined positive score (CPS) was calculated by dividing the number of PD-L1–positive cells (tumor cells, lymphocytes, and macrophages) by the total tumor cell count and multiplying by 100. pCR was defined as ypT0/is ypN0, representing no residual invasive carcinoma in the breast and sampled lymph nodes regardless of the presence of in situ disease, based on the final surgical pathology report [10,29].

### 2.3. Skeletal Muscle Characteristics

Skeletal muscle measurements were derived from abdominopelvic CT scans performed at diagnosis. At the level of the third lumbar vertebra (L3), muscle segmentation was performed using commercially available deep learning-based software (ClariMetabo version 1.03; ClariPi, Seoul, Republic of Korea) [17,30]. The software applies a two-step deep learning pipeline consisting of automated vertebral level localization (reported accuracy 99.1%) followed by 2D U-Net-based segmentation of abdominal muscle and fat components (reported Dice similarity coefficient 0.96–0.98). Segmentation was fully automated without manual correction.

Skeletal muscle tissue was defined within an attenuation range of −29 to +150 Hounsfield units (HU). Skeletal muscle area (SMA, cm^2^) was calculated as the sum of low-attenuation muscle area (−29 to 29 HU) and normal-attenuation muscle area (30 to 150 HU). It is a validated proxy for whole-body muscle mass [31]. SMD (HU) was defined as the mean attenuation value of the segmented skeletal muscle area. The skeletal muscle index (SMI, cm^2^/m^2^) was calculated by normalizing SMA to height squared, and body mass index (BMI, kg/m^2^) was determined from patient weight and height. Further information regarding the abdominal CT acquisition protocol is available in the Supplementary Method S1.

### 2.4. MRI Acquisition

Breast MRI examinations were performed using a single 3-T system (MAGNETOM Vida, Siemens Healthineers, Erlangen, Germany), with patients in the prone position using an 18-channel dedicated bilateral breast coil. The protocol included axial T2-weighted imaging, DWI, and fat-suppressed DCE T1-weighted imaging acquired using a 3D VIBE sequence. Gadobutrol (Gadovist; Bayer AG, Berlin, Germany) was administered intravenously at 0.1 mmol/kg. An ultrafast DCE acquisition began 10 s after contrast administration and continued for 1 min 25 s with a temporal resolution of 4.18 s, followed by five conventional dynamic acquisitions beginning at 93, 176, 259, 342, and 425 s. Maximum intensity projection and subtraction images were generated for each postcontrast phase using Syngo MR XA31 (Siemens Healthineers, Erlangen, Germany). Detailed acquisition and post-processing parameters are provided in Supplementary Method S2.

### 2.5. Tumor Segmentation and Radiomic Feature Extraction

Radiomic analysis was performed on the initial standard post-contrast T1-weighted images obtained from the DCE-MRI protocol (acquired at 93 s on the Vida scanner). Image preprocessing included z-score normalization of voxel intensities, isotropic resampling to 1.0 × 1.0 × 1.0 mm^3^ using B-spline interpolation, and intensity discretization using a fixed bin width of 25.

Three-dimensional tumor segmentation was manually performed using EXPRESS (Philips Healthcare, Seoul, Republic of Korea), which incorporates the PyRadiomics library (version 3.1.0) for standardized feature extraction. The entire enhancing tumor volume was delineated slice by slice rather than on a single two-dimensional section. For multifocal disease, only the largest enhancing lesion was selected for analysis, irrespective of whether it presented as a mass or non-mass enhancement. Each case was assigned to and manually segmented once by one of two board-certified breast radiologists (H.S.M. and S.H.K., with 15 and 25 years of experience in breast imaging, respectively) according to a predefined institutional segmentation protocol. Independent duplicate or repeated segmentations were not performed. Cases with indistinct tumor boundaries, multifocal disease, or non-mass enhancement that posed segmentation challenges were jointly reviewed by both radiologists, and the final contours were determined by consensus.

A total of 1132 radiomic features were extracted for radiomic-feature selection and prediction model development. These included 100 original features comprising 14 shape features, 18 first-order features, 22 gray-level co-occurrence matrix features (GLCM), 16 gray-level run-length matrix features (GLRLM), 16 gray-level size-zone matrix features (GLSZM), and 14 gray-level dependence matrix features (GLDM). An additional 344 Laplacian-of-Gaussian features (LoG) were generated using four kernel sizes of 2, 3, 4, and 5 mm, and 688 wavelet features were generated using eight wavelet decompositions: HHH, HHL, HLH, HLL, LHH, LHL, LLH, and LLL.

### 2.6. Feature Selection and Model Construction

To reduce redundancy among radiomic features, hierarchical clustering was performed using average linkage based on pairwise absolute Spearman correlation coefficients, with a threshold of 0.90 for cluster formation. The clustering procedure resulted in 325 clusters, and the feature with the largest variance on the original scale was selected from each cluster, yielding 325 candidate radiomic features.

Least absolute shrinkage and selection operator (LASSO)-penalized logistic regression was subsequently performed using the 325 candidate features after standardization. The penalty parameter λ was selected by 10-fold cross-validation using the value that minimized the mean cross-validated deviance. A radiomic score was calculated for each patient as the linear predictor from the LASSO model including the intercept and the coefficients of the selected standardized radiomic features.

Clinicopathologic and skeletal muscle predictors were selected separately. Candidate clinicopathologic variables were evaluated using stepwise logistic regression to construct the clinicopathologic model, whereas skeletal muscle variables were evaluated in a separate stepwise logistic regression model. For both procedures, the entry and retention criteria were *p* < 0.20 and *p* < 0.05, respectively. PD-L1 CPS was unavailable in 52 of 174 patients (29.9%) and was therefore excluded from primary multivariable model development. Its potential influence was evaluated in additional complete-case sensitivity analyses among the 122 patients with available PD-L1 data and separately within the NACI subgroup. Multicollinearity among predictors was assessed using pairwise Spearman correlation coefficients and variance inflation factors (VIFs), with VIF values ≥ 5 considered indicative of relevant multicollinearity.

Six candidate models were constructed to assess the incremental predictive contributions of radiomic, clinicopathologic, and skeletal muscle features: Model 1, the radiomics model; Model 2, the clinicopathologic model; Model 3, the skeletal muscle model; Model 4, the combined radiomics and clinicopathologic model; Model 5, the combined radiomics and skeletal muscle model; and Model 6, the combined radiomics, clinicopathologic, and skeletal muscle model.

### 2.7. Statistical Analysis

Categorical variables were summarized as frequencies and percentages. Continuous variables were summarized as means with standard deviations for normally distributed variables and medians with interquartile ranges for non-normally distributed variables. Group comparisons were performed using the chi-square test or Fisher’s exact test for categorical variables and Student’s t-test or the Wilcoxon rank-sum test for continuous variables, as appropriate. Univariable and multivariable Firth penalized logistic regression analyses were performed to evaluate predictors of pCR, and odds ratios (ORs) with 95% confidence intervals (CIs) were calculated. Because supervised radiomic-feature selection and model fitting used the same cohort, the radiomic-score ORs, CIs, and *p*-values do not account for uncertainty introduced by feature selection. These results were therefore interpreted as exploratory rather than confirmatory.

The apparent performance of the six candidate models was evaluated in the original dataset using the area under the receiver operating characteristic curve (AUC) for discrimination [32,33], the Hosmer–Lemeshow test for calibration, and the Brier score for overall prediction error. AUCs were compared using the DeLong test [34]. These apparent AUC comparisons were considered exploratory because they did not account for supervised predictor selection in the same dataset.

For the final model, apparent calibration was additionally assessed using a calibration plot, calibration intercept, and calibration slope. The incremental contribution of SMD was assessed by comparing Model 5 with Model 1 and Model 6 with Model 4 using differences in AUC, likelihood-ratio tests, Akaike information criterion (AIC), Brier score, integrated discrimination improvement (IDI), and continuous net reclassification improvement (NRI). The final prediction model was selected based on the highest apparent AUC, with consideration of clinical relevance. Likelihood-ratio chi-square statistics were calculated from the AIC differences between nested Firth penalized logistic regression models, with *p*-values derived from the chi-square distribution with 1 degree of freedom. These comparisons were interpreted cautiously and were not considered sufficient evidence of an incremental predictive contribution of SMD.

Internal validation was performed using 1000 bootstrap resamples. The 325 representative radiomic features obtained through outcome-independent hierarchical clustering were kept fixed, and the entire supervised modeling pipeline was repeated within each bootstrap resample. This included LASSO regression and reconstruction of the radiomic score, separate selection of clinicopathologic and skeletal muscle predictors, construction of Models 1–6, and selection of the model with the highest apparent AUC. Each bootstrap-derived selected model was evaluated in both the bootstrap sample and the original dataset. Optimism-corrected estimates of the AUC, calibration intercept, calibration slope, and Brier score were obtained using Harrell’s optimism correction procedure, based on the mean optimism estimated across the 1000 bootstrap resamples. For the calibration slope, optimism was calculated as the slope estimated in each bootstrap sample minus the slope obtained when the corresponding bootstrap-derived model was applied to the original dataset; mean optimism was subtracted from the apparent slope. Optimism-corrected AUCs were additionally calculated for Models 2 and 4 using the same bootstrap framework, repeating the selection steps applicable to each model. Radiomic-feature selection frequencies, final-model selection frequencies, and predictor inclusion frequencies in the selected final models were also recorded.

A nomogram was constructed from the final prediction model as an exploratory visualization of the model-derived pCR probabilities under the recorded treatment context. Exploratory decision curve analysis (DCA) compared the apparent net benefit of the model with comparator models and the treat-all and treat-none strategies across a range of threshold probabilities. No specific downstream clinical action was prespecified for these thresholds.

Additional analyses examined the association between treatment type and the radiomic score using Spearman correlation and evaluated the association between the radiomic score and pCR separately within the NACI and NAC groups. An exploratory treatment-by-SMD interaction term was added to Model 6. Treatment-stratified analyses also evaluated the association between SMD and pCR in the NACI and NAC groups, adjusting for the radiomic score and Ki-67. Complete-case sensitivity analyses added PD-L1 CPS to Model 6 as either a continuous variable (Model 7-1) or a categorical variable (CPS ≥ 10 vs. <10; Model 7-2), with additional analyses restricted to NACI-treated patients with available PD-L1 data.

All statistical analyses were conducted using SAS (version 9.4; SAS Institute, Cary, NC, USA) and R (version 4.6.1; R Foundation for Statistical Computing, Vienna, Austria), including the cluster and glmnet packages. A two-sided *p*-value < 0.05 was considered statistically significant.

## 3. Results

### 3.1. Baseline Characteristics

A total of 174 patients (mean age, 52.0 ± 11.1 years) with TNBC who underwent neoadjuvant treatment followed by surgery were included in the final analysis (Table 1). Of these patients, 94 (54.0%) received NACI and 80 (46.0%) received NAC. Based on the final surgical pathology, 85 patients (48.9%) achieved pCR, whereas 89 (51.1%) did not achieve pCR. The pCR rate was significantly greater in the NACI group compared to the NAC group (59.6% [56/94] vs. 36.3% [29/80], *p* = 0.002).

Patients aged ≥ 40 years represented 87.4% (152/174) of the entire cohort. There was no significant difference in the distribution of age, menopausal status, or family history of breast cancer between the pCR and non-pCR groups (all *p* > 0.05). Clinical T stage, clinical N stage, and overall clinical TNM stage also did not differ significantly between the groups (all *p* > 0.05). Within the total cohort, the most commonly observed characteristics were clinical T2 (75.3%, 131/174), clinical N1 (43.1%, 75/174), and stage II disease (62.1%, 108/174).

The majority of tumors were invasive ductal carcinoma (96.0%, 167/174), with no statistical difference in proportion between the groups (*p* = 0.444). Tumor grade was significantly higher in the pCR group, with grade III cases being more prevalent compared to the non-pCR group (85.9% [73/85] vs. 62.9% [56/89], *p* = 0.001). The median Ki-67 index for the whole cohort was 59.9 (IQR, 40.0–75.4), and as a continuous variable, the index was significantly greater in the pCR group (67.0 [IQR, 48.0–80.0] vs. 53.0 [IQR, 34.0–67.0], *p* < 0.001). Nevertheless, dichotomizing at a 20% threshold revealed no significant difference between pCR and non-pCR groups (*p* = 0.139). PD-L1 CPS data were available for 122 patients (70.1%), with a median value of 10.0 (IQR, 5.0–20.0) for the cohort. Both continuous and categorical analyses (cutoff ≥ 10) indicated significantly elevated values for PD-L1 CPS in the pCR group relative to the non-pCR group (continuous: 15.0 [10.0–30.0] vs. 10.0 [5.0–10.0], *p* = 0.001; categorical: 80.3% [49/61] vs. 57.4% [35/61], *p* = 0.006).

For the entire cohort, the mean SMA was 100.2 ± 15.4 cm^2^, with no significant difference between the pCR and non-pCR groups (*p* = 0.170). Similarly, SMI showed no significant association with pCR, whether analyzed as a continuous variable or dichotomized using the previously reported cutoff of 38.5 [25] or the cohort median value of 39.5 (all *p* > 0.05). BMI was also not significantly different between the two groups (*p* = 0.117). In contrast, SMD was significantly higher in the pCR group than in the non-pCR group (median 50.0 [45.0–54.0] vs. 48.0 [42.0–52.0], *p* = 0.006).

### 3.2. Radiomic Score Construction

From the initial 1132 extracted radiomic features, hierarchical clustering was performed to reduce redundancy, yielding 325 representative features (26 original, 84 LoG-transformed, and 215 wavelet features). Using these features, LASSO regression with 10-fold cross-validation selected 7 features with nonzero coefficients, which were combined to construct the final radiomic score. The selected radiomic features and their corresponding coefficients are summarized in Supplementary Table S1. The radiomic score for predicting pCR was calculated as follows:

Radiomic score = −0.0463

−0.0513 × log_sigma_4_0_mm_3D_glszm_SizeZoneNonUniformity

−0.0674 × log_sigma_5_0_mm_3D_glszm_GrayLevelNonUniformity

+ 0.0564 × wavelet_LHL_glszm_GrayLevelNonUniformityNormalized

−0.0143 × wavelet_HLL_glcm_Correlation

−0.0896 × wavelet_HLL_glcm_ClusterTendency

+ 0.1120 × wavelet_HHH_firstorder_Mean

−0.0071 × wavelet_LLL_glszm_SmallAreaLowGrayLevelEmphasis

Each radiomic feature in this equation represents its standardized value, as used in LASSO model fitting. The resulting radiomic score was incorporated as a continuous predictor in Model 1 and the integrated models (Models 4–6).

### 3.3. Clinicopathologic and Skeletal Muscle Predictors of pCR

In the univariable analysis, NACI (vs. NAC; OR = 2.56, 95% CI: 1.39–4.73, *p* = 0.003), tumor grade III (OR = 3.49, 95% CI: 1.66–7.33, *p* = 0.001), elevated Ki-67 (OR = 1.03, 95% CI: 1.01–1.04, *p* < 0.001), and PD-L1 CPS (continuous: OR = 1.03, 95% CI: 1.01–1.06, *p* = 0.013; CPS ≥ 10 vs. <10: OR = 2.96, 95% CI: 1.32–6.63, *p* = 0.009) were significantly associated with pCR (Table 2). Because PD-L1 CPS was available for 122 of 174 patients (70.1%), it was excluded from the primary multivariable models and evaluated in complete-case sensitivity analyses described in Section 3.7. In the primary clinicopathologic multivariable analysis, NACI (adjusted OR = 2.34, 95% CI: 1.24–4.41, *p* = 0.009) and Ki-67 (adjusted OR = 1.02, 95% CI: 1.01–1.04, *p* = 0.001) remained independent predictors and were included in Model 2.

Among the skeletal muscle parameters, SMD was retained as the sole predictor in the skeletal muscle model (Model 3; OR per 1-HU increase = 1.06, 95% CI: 1.02–1.11, *p* = 0.005). Other parameters, including SMA, continuous SMI, low-SMI status, and BMI, were not significantly associated with pCR in the univariable analyses.

### 3.4. Integrated Models and Apparent Performance

The radiomic-score OR per SD increase was 2.93 (95% CI: 1.86–4.63, *p* < 0.001) in Model 1, and its regression coefficient was positive in all integrated models (Table 3). With chemotherapy as the reference category, the regression coefficient for NACI remained positive in Models 2, 4, and 6 (β = 0.85, 0.68, and 0.59, respectively). The corresponding adjusted ORs were 2.34 (95% CI: 1.24–4.41, *p* = 0.009), 1.97 (95% CI: 0.99–3.91, *p* = 0.054), and 1.81 (95% CI: 0.90–3.64, *p* = 0.095). SMD was significantly associated with pCR in Model 5 (adjusted OR = 1.05, 95% CI: 1.00–1.09, *p* = 0.045), but not in Model 6 (adjusted OR = 1.04, 95% CI: 0.99–1.09, *p* = 0.130). No relevant multicollinearity was identified in Models 4 and 6 (VIF range, 1.02–1.08; Supplementary Table S2).

**Figure 2:**
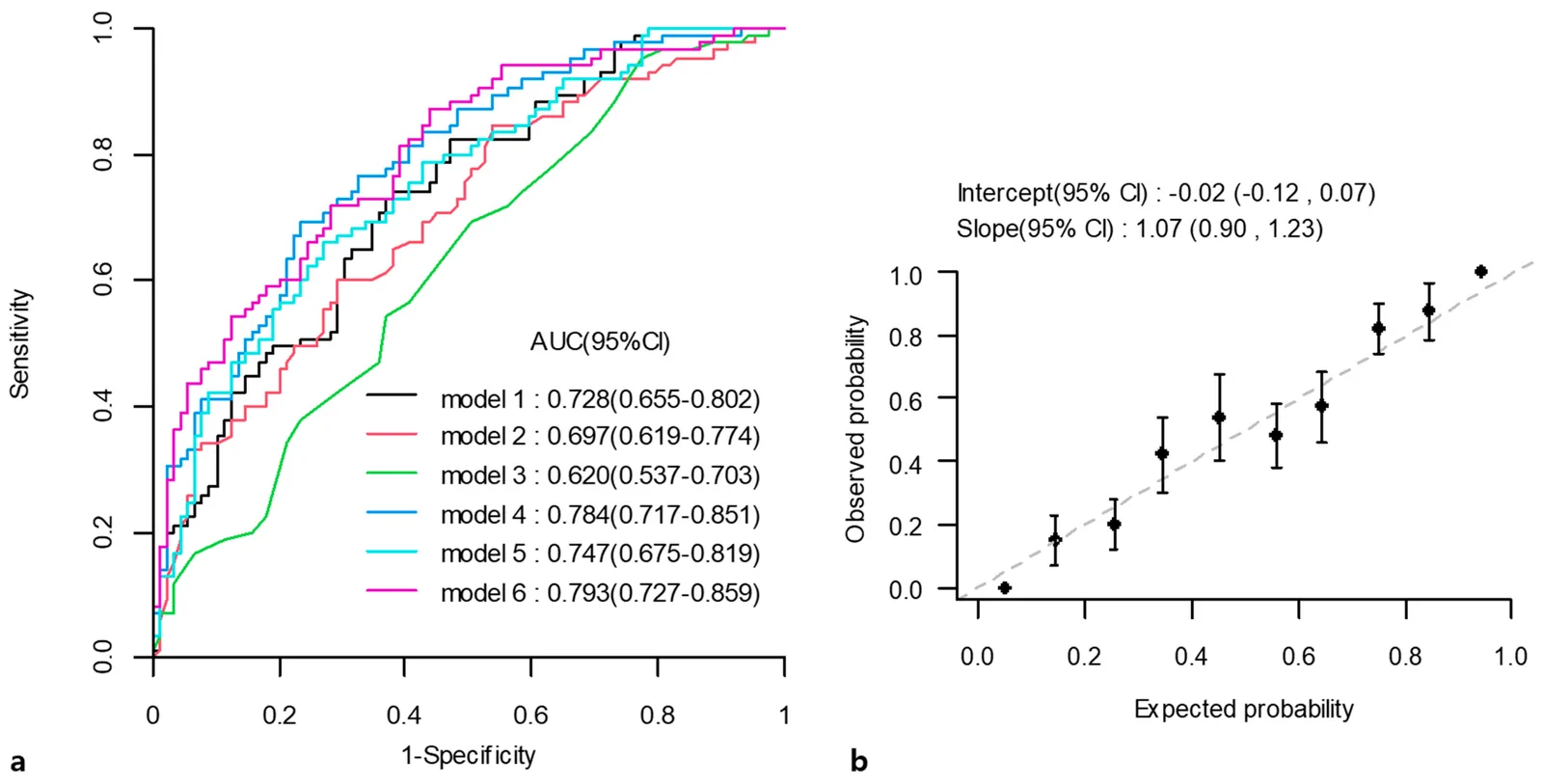
Apparent performance of models for predicting pathologic complete response. (**a**) Receiver operating characteristic (ROC) curves of the six candidate models in the original dataset. (**b**) Apparent calibration plot of Model 6 in the original dataset. The dashed diagonal line represents perfect calibration. Points represent observed pCR proportions within groups of patients with similar predicted probabilities, and vertical bars indicate 95% confidence intervals. AUC = area under the curve; CI = confidence interval.

The apparent performance of the six candidate models is summarized in Table 4 and Figure 2a. Model 2, with an AUC of 0.697 (95% CI: 0.619–0.774), served as the reference model. Model 4 achieved an apparent AUC of 0.784 (95% CI: 0.717–0.851; exploratory DeLong comparison with Model 2, *p* = 0.008). Model 6 had the highest apparent AUC of 0.793 (95% CI: 0.727–0.859; exploratory DeLong comparison with Model 2, *p* = 0.005). No significant lack of fit was detected by the Hosmer–Lemeshow test for any model (all *p* > 0.05).

The incremental contribution of SMD was evaluated in two nested model comparisons (Supplementary Table S3). Adding SMD to the radiomics-only model (Model 5 vs. Model 1) reduced AIC from 209.533 to 199.509 and the apparent Brier score from 0.209 to 0.203, with an AIC-derived likelihood-ratio *p*-value < 0.001. However, the increase in AUC was not statistically significant (ΔAUC = 0.019, 95% CI: −0.015 to 0.053, *p* = 0.274). Adding SMD to the radiomics-plus-clinicopathologic model (Model 6 vs. Model 4) reduced AIC from 186.275 to 178.284 and the apparent Brier score from 0.190 to 0.187 with an AIC-derived likelihood-ratio *p*-value of 0.002. The corresponding increase in AUC was small and not statistically significant (ΔAUC = 0.009, 95% CI: −0.013 to 0.030, *p* = 0.428). IDI was significant only for Model 5 versus Model 1 (0.022, 95% CI: 0.000–0.044, *p* = 0.047), whereas continuous NRI was not significant in either comparison. Model 6 was selected as the final model according to the AUC-based selection criterion described in Section 2.

### 3.5. Bootstrap Internal Validation and Selection Stability

In the original dataset, Model 6 had an apparent calibration slope of 1.07 (95% CI: 0.90–1.23) and a calibration intercept of −0.02 (95% CI: −0.12 to 0.07; Figure 2b). The Hosmer–Lemeshow test yielded *p* = 0.557, and the apparent Brier score was 0.187 (95% CI: 0.158–0.217).

After internal validation using 1000 bootstrap resamples, the optimism-corrected AUC was 0.629 (95% CI: 0.500–0.701). The optimism-corrected calibration intercept, calibration slope, and Brier score were −0.024 (95% CI: −0.096 to 0.061), 0.871 (95% CI: −0.512 to 1.068), and 0.309 (95% CI: 0.234–0.395), respectively. The corrected Brier point estimate exceeded the approximately 0.250 Brier score for constant prediction at the observed pCR prevalence (85/174). The additionally calculated optimism-corrected AUCs were 0.659 for Model 2 and 0.622 for Model 4. Thus, the higher apparent AUCs of Models 4 and 6 were not maintained after optimism correction.

Radiomic-feature selection varied across bootstrap resamples. Three of the seven features constituting the original radiomic score were selected in more than 80% of resamples: wavelet_HHH_firstorder_Mean (93.9%), wavelet_LHL_glszm_GrayLevelNonUniformityNormalized (87.3%), and log_sigma_4_0_mm_3D_glszm_SizeZoneNonUniformity (80.7%). Selection frequencies for the remaining four original-score features were 36.6%, 32.4%, 22.9%, and 15.1%, respectively (Supplementary Table S4).

Model 6 was selected most frequently as the final model (50.3%), followed by Model 4 (30.2%), Model 1 (10.7%), Model 5 (8.7%), Model 2 (0.1%), and Model 3 (0.0%) (Supplementary Table S5). Models containing radiomic information were selected in 99.9% of resamples. Among the selected final models, the inclusion frequencies were 99.9% for the radiomic score, 60.3% for Ki-67, 55.8% for SMD, and 50.8% for treatment type (Supplementary Table S6).

### 3.6. Nomogram Construction and Exploratory Decision Curve Analysis

**Figure 3:**
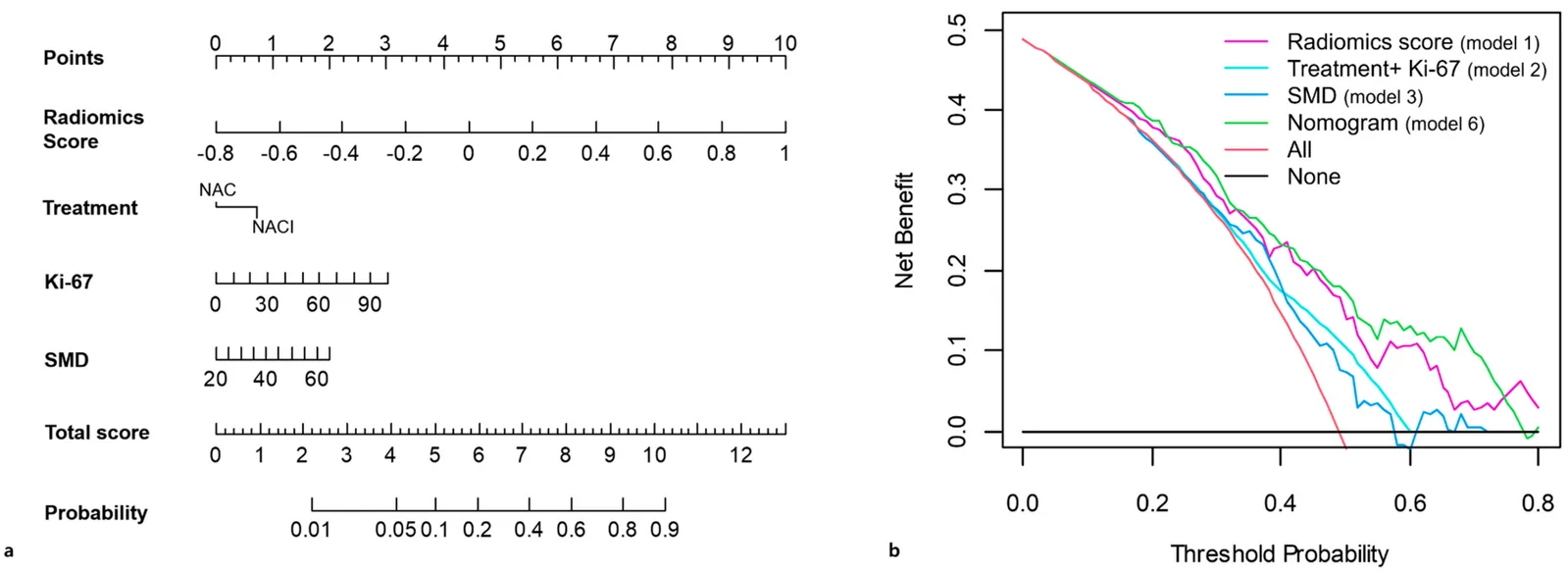
Nomogram and exploratory decision curve analysis (DCA) of the final model (Model 6). (**a**) Nomogram incorporating radiomic score, recorded treatment, Ki-67, and skeletal muscle density (SMD) for estimating pathologic complete response probability. The nomogram is an exploratory visualization and has not been validated for clinical decision-making. (**b**) Exploratory DCA comparing the apparent net benefit of the nomogram with the comparator models and conventional treat-all and treat-none strategies. The treat-all and treat-none curves represent applying a hypothetical downstream action to all or no patients, respectively; they do not represent assigning all patients to NACI or NAC. No specific clinical action was prespecified for the evaluated thresholds. NACI = neoadjuvant chemoimmunotherapy; NAC = neoadjuvant chemotherapy.

A nomogram incorporating the radiomic score, recorded treatment type, Ki-67, and SMD was constructed from Model 6 as an exploratory visualization of model-derived pCR probabilities under the recorded treatment context (Figure 3a). The model coefficients, predictor coding, and probability equation are provided in Supplementary Table S7.

Exploratory DCA showed a higher apparent net benefit for the nomogram than for the comparator models and the treat-all and treat-none strategies within several threshold intervals: approximately 10–22%, 26–32%, 34–40%, and 42–74% (Figure 3b). These intervals describe the observed comparative net benefit and were not prespecified as clinical decision thresholds.

### 3.7. Additional Sensitivity Analyses

With treatment coded as NACI = 1 and NAC = 0, a weak positive correlation was observed between treatment type and the radiomic score (ρ = 0.19, 95% CI: 0.05–0.33). The median radiomic score was higher in the NACI group than in the NAC group (−0.02 vs. −0.09, *p* = 0.010). In treatment-stratified analyses, the radiomic score was positively associated with pCR in both the NACI group (OR per SD increase = 2.53, 95% CI: 1.39–4.63; AUC = 0.692) and the NAC group (OR per SD increase = 2.79, 95% CI: 1.45–5.37; AUC = 0.748) (Supplementary Tables S2 and S8).

In treatment-stratified analyses adjusted for the radiomic score and Ki-67, SMD was associated with pCR in the NACI group (*n* = 94; OR per 1-HU increase, 1.08; 95% CI: 1.01–1.16; *p* = 0.026), whereas no significant association was observed in the NAC group (*n* = 80; OR, 1.00; 95% CI: 0.94–1.06; *p* = 0.993). The treatment-by-SMD interaction in Model 6 was not statistically significant (*p* = 0.082), providing insufficient evidence of a difference in the SMD–pCR association between treatment groups.

PD-L1 CPS was available for 122 patients and missing for 52 (29.9%). PD-L1 missingness differed significantly according to operation year (χ^2^ = 38.92, *p* < 0.001), with missing data particularly frequent in 2021 and less frequent in subsequent years. Patients with and without available PD-L1 data differed in treatment distribution (NACI, 66.4% vs. 25.0%, *p* < 0.001), but not in pCR outcome, age, clinical T or N category, clinical stage, Ki-67, SMD, or radiomic score (all *p* > 0.05) (Supplementary Table S9).

In the complete-case cohort, the radiomic score showed a positive association with pCR after adding PD-L1 CPS to Model 6 as either a continuous variable (Model 7-1; adjusted OR per SD increase = 1.85, 95% CI: 1.15–2.97, *p* = 0.011) or a categorical variable (Model 7-2; adjusted OR per SD increase = 1.88, 95% CI: 1.16–3.05, *p* = 0.011). PD-L1 CPS did not reach statistical significance in either formulation (continuous CPS, *p* = 0.084; CPS ≥ 10 vs. <10, *p* = 0.230). Adding PD-L1 CPS produced small, nonsignificant increases in AUC compared with Model 6 fitted in the same complete-case cohort (ΔAUC = 0.016, *p* = 0.343; and ΔAUC = 0.007, *p* = 0.498, respectively) (Supplementary Table S10 and Figure S1).

Among the 81 NACI-treated patients with available PD-L1 data, the radiomic score showed a positive association with pCR in models incorporating either continuous or categorical PD-L1 CPS (adjusted OR per SD increase = 1.89, *p* = 0.033; and 1.96, *p* = 0.027, respectively). PD-L1 CPS was not significantly associated with pCR in either model (*p* = 0.147 and *p* = 0.418, respectively) (Supplementary Table S11).

## 4. Discussion

This study investigated the predictive contributions of pretreatment MRI radiomics, clinicopathologic factors, and skeletal muscle measures to pCR in patients with TNBC undergoing neoadjuvant therapy. Among the six evaluated models, Model 6, incorporating the radiomic score, treatment type, Ki-67, and skeletal muscle density (SMD), achieved the highest apparent AUC of 0.793. These apparent findings support further investigation of combined imaging and clinical information but do not establish improved predictive performance after optimism correction. Notably, radiomic information was retained in 99.9% of bootstrap-selected models, supporting its potential relevance to response prediction.

Nevertheless, bootstrap internal validation revealed substantial optimism in the apparent predictive performance, with the optimism-corrected AUC decreasing to 0.629. Although Model 6 was the most frequently selected model, it was retained in only 50.3% of bootstrap resamples, and the selection frequencies of the seven original radiomic features varied considerably (15.1–93.9%). Only three of the seven original features were selected in more than 80% of resamples, indicating that the composition of the radiomic score was sensitive to resampling. These selection frequencies describe sampling stability and do not establish feature reproducibility with respect to segmentation variability. Calibration and overall prediction error also warranted caution after optimism correction, with a calibration slope of 0.871 (95% CI: −0.512 to 1.068) and a Brier score of 0.309, exceeding the approximately 0.250 benchmark for constant prediction at the observed pCR prevalence. These findings highlight the instability of feature and model selection and indicate that external validation is necessary before clinical application. The corrected AUCs of Models 2, 4, and 6 were 0.659, 0.622, and 0.629, respectively, so the apparent advantage of the radiomics-containing models was not supported by the corrected AUC estimates.

In Model 6, the radiomic-score OR per SD increase was 2.53 (95% CI, 1.62–3.96). However, because the score was selected and weighted using the same cohort, this estimate is exploratory and should not be interpreted as confirmatory evidence of an independent association or a validated effect magnitude. In the present study, radiomic features were extracted from the intratumoral region on DCE-MRI to characterize tumor enhancement patterns [35,36]. Furthermore, prior research indicated that performing analysis within the intratumoral region alone achieved predictive accuracy similar to or exceeding that of peritumoral or combined feature sets, with AUCs reported between 0.713 and 0.749 [37].

Consistent with earlier studies, our results provide additional evidence for radiomics-based response prediction. A pre-NAC DCE-MRI study reported an AUC of 0.75, increasing to 0.811 after incorporating estrogen receptor status [38], while a TNBC ensemble model improved from an AUC of 0.749 for radiomics alone to 0.802 after adding clinicopathologic and conventional MRI information [37]. In our cohort, the apparent AUC was 0.728 for the radiomics-only model and 0.793 for Model 6. The NACI coefficient remained positive after adjustment for the radiomic score, and the score was positively associated with pCR within both treatment strata. A weak positive correlation between treatment type and the radiomic score was also observed. These within-cohort findings should be interpreted alongside the lower optimism-corrected performance and do not constitute independent validation of the radiomic score.

Recent research has expanded beyond tumor-specific variables to examine body composition in breast cancer [39]. SMD reflects intramuscular fat infiltration and muscle quality and may provide information distinct from muscle quantity [40,41,42,43]. In our cohort, SMD was associated with pCR in the muscle-only model and remained significant when added to radiomics alone, but not in the fully integrated Model 6 (*p* = 0.130). The AIC-derived likelihood-ratio results were interpreted cautiously because their discrepancy with the coefficient-specific regression results was not fully resolved; these results alone do not establish an incremental predictive contribution of SMD. In particular, adding SMD to the radiomics-plus-clinicopathologic model increased the AUC by only 0.009 (*p* = 0.428). IDI was significant only for Model 5 versus Model 1, and continuous NRI was nonsignificant in both comparisons. SMD was included in 55.8% of the selected final models during bootstrap resampling. In exploratory analyses, SMD was associated with pCR in the NACI group, but the treatment-by-SMD interaction was not statistically significant (*p* = 0.082). Given the cohort overlap with our previous study [17], these findings require independent confirmation. Overall, the incremental predictive contribution of SMD beyond radiomic and clinicopathologic information remains uncertain.

Nomograms can summarize multiple clinical and imaging predictors as individualized probabilities [44,45,46,47,48,49]. In the present study, exploratory DCA indicated higher apparent net benefit than selected alternatives across parts of the evaluated threshold range. However, because treatment type is included as a predictor and the outcome is observed after treatment, the model estimates pCR probability conditional on the recorded treatment context and does not estimate counterfactual benefit from choosing one treatment over another. No specific downstream clinical action was prespecified for the evaluated thresholds. The observed intervals therefore should not be interpreted as recommended clinical decision thresholds or as evidence of established clinical utility. Accordingly, the nomogram should be regarded as an exploratory visualization of Model 6 and its model-derived probabilities under the recorded treatment context, rather than as a basis for treatment selection, escalation, or de-escalation, or as a replacement for established clinical assessment. External validation and prospective clinical evaluation are required before the nomogram can be used to guide treatment decisions.

Several limitations should be acknowledged. First, this was a single-center retrospective study with a relatively small sample. Although bootstrap internal validation repeated the supervised radiomic-feature selection, clinicopathologic and skeletal muscle predictor selection, and final-model selection procedures, the outcome-independent reduction from 1132 to 325 representative radiomic candidates was kept fixed. The validation estimates were therefore conditional on this initial feature-reduction step. The substantial decline in discrimination after optimism correction and the variability in feature and model selection limit confidence in the reproducibility and generalizability of the developed model. External validation is required. Second, all MRI examinations were acquired using a single scanner and protocol, which minimized within-cohort acquisition heterogeneity but may limit generalizability to other scanner platforms and institutions. External multicenter and multiscanner validation is therefore required. Third, treatment allocation was nonrandomized in this retrospective cohort. Although treatment type was included as a predictor, residual confounding related to treatment allocation cannot be excluded. In addition, PD-L1 data were missing in 29.9% of patients, and availability varied by treatment group and operation year. Individual reasons for non-testing could not be retrospectively ascertained. The complete-case sensitivity analyses, including those restricted to NACI-treated patients with available PD-L1 data, may therefore be subject to selection bias and cannot fully address the influence of potentially nonrandom missingness. Fourth, radiomic features were derived from a single-phase MRI sequence. Manual tumor segmentation was performed once per case by one of two radiologists without independent duplicate or repeated segmentation. Therefore, interobserver and intraobserver reproducibility of the tumor contours, derived radiomic features, and radiomic score could not be formally assessed. Although a standardized protocol and consensus review were used for cases with uncertain boundaries, observer-related variability and its effect on model performance cannot be excluded. Fifth, DCA was exploratory and was evaluated in the original dataset without external validation. Because no specific clinical action was prespecified for a given pCR probability threshold, its findings should not be interpreted as evidence of established clinical utility or as support for treatment selection. Finally, the incremental predictive contribution of SMD was small and varied across model specifications, requiring confirmation in larger independent cohorts.

## 5. Conclusions

In conclusion, pretreatment MRI radiomics showed exploratory associations with pCR in this cohort of patients with TNBC undergoing neoadjuvant therapy. The integrated model incorporating the radiomic score, treatment type, Ki-67, and skeletal muscle density achieved the highest apparent discrimination among the evaluated models. However, the substantial reduction in discrimination after bootstrap optimism correction and variability in feature and model selection indicate that its predictive performance requires further validation. The apparent performance gains were not supported by the corrected AUC estimates, and the incremental predictive contribution of SMD remains uncertain; external validation is required before clinical application.

## Figures and Tables

**Table 1:** Clinicopathologic and skeletal muscle characteristics.

	Total (*n* = 174)	pCR (*n* = 85)	Non-pCR (*n* = 89)	*p* Value
Clinicopathologic characteristics				
Neoadjuvant treatment				0.002
Chemoimmunotherapy	94 (54.0)	56 (65.9)	38 (42.7)	
Chemotherapy	80 (46.0)	29 (34.1)	51 (57.3)	
Age	52.0 ± 11.1	50.7 ± 11.1	53.3 ± 11.0	0.118
<40	22 (12.6)	15 (17.6)	7 (7.9)	0.052
≥40	152 (87.4)	70 (82.4)	82 (92.1)	
Menopausal status at diagnosis				0.224
Postmenopause	86 (49.4)	38 (44.7)	48 (53.9)	
Premenopause	88 (50.6)	47 (55.3)	41 (46.1)	
FHBC				0.448
No	150 (86.2)	75 (88.2)	75 (84.3)	
Yes	24 (13.8)	10 (11.8)	14 (15.7)	
BRCA1/2 mutation				0.923
Absent	158 (90.8)	77 (90.6)	81 (91.0)	
Present	16 (9.2)	8 (9.4)	8 (9.0)	
Clinical T				0.636
T1	5 (2.9)	3 (3.5)	2 (2.2)	
T2	131 (75.3)	67 (78.8)	64 (71.9)	
T3	33 (19.0)	13 (15.3)	20 (22.5)	
T4	5 (2.9)	2 (2.4)	3 (3.4)	
Clinical N				0.940
N0	46 (26.4)	22 (25.9)	24 (27.0)	
N1	75 (43.1)	38 (44.7)	37 (41.6)	
N2	23 (13.2)	10 (11.8)	13 (14.6)	
N3	30 (17.2)	15 (17.6)	15 (16.9)	
Clinical TNM stage				0.940
II	108 (62.1)	53 (62.4)	55 (61.8)	
III	66 (37.9)	32 (37.6)	34 (38.2)	
Tumor histology				0.444
Invasive ductal carcinoma	167 (96.0)	83 (97.6)	84 (94.4)	
Others (Invasive lobular carcinoma, etc)	7 (4.0)	2 (2.4)	5 (5.6)	
Tumor grade				0.001
I-II	45 (25.9)	12 (14.1)	33 (37.1)	
III	129 (74.1)	73 (85.9)	56 (62.9)	
Ki-67	59.9 (40.0–75.4)	67.0 (48.0–80.0)	53.0 (34.0–67.0)	<0.001
<20	11 (6.3)	3 (3.5)	8 (9.0)	0.139
≥20	163 (93.7)	82 (96.5)	81 (91.0)	
PD-L1 CPS (*n* = 122)	10.0 (5.0–20.0)	15.0 (10.0–30.0)	10.0 (5.0–10.0)	0.001
<10	38 (31.1)	12 (19.7)	26 (42.6)	0.006
≥10	84 (68.9)	49 (80.3)	35 (57.4)	
Skeletal muscle characteristics				
Skeletal muscle area (SMA, cm^2^)	100.2 ± 15.4	98.6 ± 17.2	101.8 ± 13.4	0.170
Skeletal muscle index (SMI, cm^2^/m^2^)	39.8 ± 5.9	39.4 ± 6.7	40.2 ± 5.0	0.377
Low SMI (≤38.5 cm^2^/m^2^)	78 (44.8)	41 (48.2)	37 (41.6)	0.377
Normal SMI (>38.5 cm^2^/m^2^)	96 (55.2)	44 (51.8)	52 (58.4)	
<median (<39.5)	86 (49.4)	46 (54.1)	40 (44.9)	0.226
≥median (≥39.5)	88 (50.6)	39 (45.9)	49 (55.1)	
Skeletal muscle density (HU)	48.0 (44.0–54.0)	50.0 (45.0–54.0)	48.0 (42.0–52.0)	0.006
Body mass index (BMI, kg/m^2^)	23.2 (21.0–26.0)	22.4 (20.9–25.1)	23.6 (21.5–26.7)	0.117

pCR = pathologic complete response; FHBC = family history of breast cancer; BRCA1/2 = breast cancer gene 1/2; T = tumor stage; N = nodal stage; TNM = tumor–node–metastasis; PD-L1 CPS = programmed death-ligand 1 combined positive score. Data are presented as *n* (%), mean ± standard deviation, or median (interquartile range). Percentages in the pCR and non-pCR columns are column percentages. Treatment-specific pCR rates were 59.6% (56/94) in the NACI group and 36.3% (29/80) in the NAC group. *p* values were calculated using the chi-square test, Fisher’s exact test, Student’s t-test, or Wilcoxon rank-sum test, as appropriate.

**Table 2:** Logistic regression analyses for predicting pCR.

	Univariable		Multivariable	
	OR (95% CI)	*p* Value	Adjusted OR (95% CI)	*p* Value
Clinicopathologic characteristics			Clinicopathologic model	
Neoadjuvant treatment				
Chemoimmunotherapy	2.56 (1.39–4.73)	0.003	2.34 (1.24–4.41)	0.009
Chemotherapy	1		1	
Age, continuous	0.98 (0.95–1.01)	0.127		
Age				
<40	1			
≥40	0.41 (0.16–1.07)	0.068		
Menopausal status at diagnosis				
Postmenopause	0.69 (0.38–1.26)	0.230		
Premenopause	1			
FHBC				
No	1			
Yes	0.72 (0.30–1.73)	0.468		
BRCA1/2 mutation				
Absent	1			
Present	1.05 (0.38–2.94)	0.924		
Clinical T		0.649		
T1	1			
T2	0.75 (0.12–4.57)	0.753		
T3	0.47 (0.07–3.18)	0.439		
T4	0.51 (0.04–6.31)	0.600		
Clinical N		0.947		
N0	1			
N1	1.12 (0.54–2.33)	0.766		
N2	0.85 (0.31–2.32)	0.747		
N3	1.09 (0.43–2.73)	0.856		
Clinical TNM stage				
II	1			
III	0.98 (0.53–1.80)	0.941		
Tumor histology				
Invasive ductal carcinoma	1			
Others	0.46 (0.09–2.34)	0.350		
Tumor grade				
I-II	1			
III	3.49 (1.66–7.33)	0.001		
Ki-67, continuous	1.03 (1.01–1.04)	<0.001	1.02 (1.01–1.04)	0.001
Ki-67				
<20	1			
≥20	2.46 (0.65–9.35)	0.187		
PD-L1 CPS (*n* = 122), continuous	1.03 (1.01–1.06)	0.013		
PD-L1 CPS (*n* = 122)				
<10	1			
≥10	2.96 (1.32–6.63)	0.009		
Skeletal muscle characteristics			Skeletal muscle model	
Skeletal muscle area	0.99 (0.97–1.01)	0.186		
Skeletal muscle index (SMI), continuous	0.98 (0.93–1.03)	0.391		
Low SMI (≤38.5 cm^2^/m^2^)	1.31 (0.72–2.38)	0.383		
Normal SMI (>38.5 cm^2^/m^2^)	1			
<median (<39.5)	1.44 (0.79–2.61)	0.232		
≥median (≥39.5)	1			
Skeletal muscle density	1.06 (1.02–1.11)	0.005	1.06 (1.02–1.11)	0.005
Body mass index, continuous	0.96 (0.89–1.04)	0.346		

pCR = pathologic complete response; OR = odds ratio; CI = confidence interval; FHBC = family history of breast cancer; BRCA1/2 = breast cancer gene 1/2; T = tumor stage; N = nodal stage; PD-L1 CPS = programmed death-ligand 1 combined positive score; SMA = skeletal muscle area; SMD = skeletal muscle density; SMI = skeletal muscle index; BMI = body mass index. PD-L1 CPS was excluded from primary multivariable model development because of missing data.

**Table 3:** Odds ratios from logistic regression models for predicting pCR.

Predictor	β Coefficient ± SE	Adjusted OR (95% CI)	*p* Value
Model 1: Radiomics model			
Radiomic score, continuous	5.15 ± 1.11	2.93 (1.86–4.63)	<0.001
Model 2: Clinicopathologic model			
Chemoimmunotherapy	0.85 ± 0.32	2.34 (1.24–4.41)	0.009
Ki-67, continuous	0.02 ± 0.01	1.02 (1.01–1.04)	0.001
Model 3: Skeletal muscle model			
SMD, continuous	0.06 ± 0.02	1.06 (1.02–1.11)	0.005
Model 4: model 1 + 2			
Radiomic score, continuous	4.64 ± 1.09	2.64 (1.69–4.13)	<0.001
Chemoimmunotherapy	0.68 ± 0.35	1.97 (0.99–3.91)	0.054
Ki-67, continuous	0.03 ± 0.01	1.03 (1.01–1.04)	0.002
Model 5: model 1 + 3			
Radiomic score, continuous	4.83 ± 1.11	2.75 (1.74–4.33)	<0.001
SMD, continuous	0.04 ± 0.02	1.05 (1.00–1.09)	0.045
Model 6: model 1 + 2+3			
Radiomic score, continuous	4.45 ± 1.09	2.53 (1.62–3.96)	<0.001
Chemoimmunotherapy	0.59 ± 0.36	1.81 (0.90–3.64)	0.095
Ki-67, continuous	0.02 ± 0.01	1.02 (1.01–1.04)	0.003
SMD, continuous	0.04 ± 0.02	1.04 (0.99–1.09)	0.130

pCR = pathologic complete response; SE = standard error; OR = odds ratio; CI = confidence interval; SMD = skeletal muscle density. The ORs for Ki-67 and SMD were calculated per one-unit increase, whereas the OR for the radiomic score was calculated per one-standard-deviation increase. The β coefficients and SEs for the radiomic score are shown on the original unstandardized score scale. Chemotherapy served as the reference category for the treatment variable. Radiomic-score ORs, CIs, and *p*-values are exploratory within-cohort estimates without adjustment for supervised feature selection.

**Table 4:** Apparent performance of the six candidate models for predicting pCR.

Model	AUC (95% CI)	Hosmer–Lemeshow Test, *p* value	Brier Score (95% CI)	Difference of AUC (95% CI)	*p* Value in AUC Comparison to Model 2
Model 1: Radiomics model	0.728 (0.655–0.802)	0.104	0.209 (0.184–0.234)	0.032 (−0.071, 0.134)	0.546
Model 2: Clinicopathologic model	0.697 (0.619–0.774)	0.172	0.221 (0.198–0.244)	reference	Reference
Model 3: Skeletal muscle model	0.620 (0.537–0.703)	0.467	0.238 (0.222–0.253)	−0.077 (−0.183, 0.030)	0.157
Model 4: model 1 + 2	0.784 (0.717–0.851)	0.979	0.190 (0.161–0.219)	0.087 (0.023, 0.152)	0.008
Model 5: model 1 + 3	0.747 (0.675–0.819)	0.557	0.203 (0.176–0.229)	0.051 (−0.050, 0.151)	0.323
Model 6: model 1 + 2+3	0.793 (0.727–0.859)	0.557	0.187 (0.158–0.217)	0.096 (0.029, 0.163)	0.005

pCR = pathologic complete response; AUC = area under the curve; CI = confidence interval. Hosmer–Lemeshow test was used to assess model calibration, and the DeLong test was used to compare AUCs between models. AUC comparisons are exploratory apparent comparisons and do not account for supervised predictor selection.

## Data Availability

The datasets used and/or analyzed during the current study are available from the corresponding author upon reasonable request.

## Supplementary Materials

The following supporting information can be downloaded at https://www.mdpi.com/article/10.3390/diagnostics16213679/s1, Supplementary Method S1: Abdominopelvic CT Acquisition; Supplementary Method S2: Breast MRI Acquisition and Post-processing; Table S1: Selected radiomic features and corresponding coefficients used for radiomic score construction; Table S2: Multicollinearity diagnostics for integrated models; Table S3: Incremental predictive value of SMD; Table S4: Radiomic-feature selection frequencies across 1000 bootstrap resamples; Table S5: Final-model selection frequencies across 1000 bootstrap resamples; Table S6: Variable inclusion frequencies in selected final models across 1000 bootstrap resamples; Table S7: Regression coefficients and prediction equation for Model 6; Table S8: Treatment–radiomic score analyses; Table S9: Availability and Missingness of PD-L1 Data; Table S10: Complete-case models incorporating PD-L1 (*n* = 122); Table S11: NACI subgroup analyses among patients with available PD-L1 data (*n* = 81); Figure S1: Calibration plots for complete-case models.

## Abbreviations

The following abbreviations are used in this manuscript:

AUC	Area under the curve
DCA	Decision curve analysis
DCE-MRI	Dynamic contrast-enhanced magnetic resonance imaging
NAC	Neoadjuvant chemotherapy
NACI	Neoadjuvant chemoimmunotherapy
pCR	Pathologic complete response
PD-L1	Programmed death-ligand 1
SMD	Skeletal muscle density
SMI	Skeletal muscle index
TNBC	Triple-negative breast cancer
