# Supplementary material for: MRI Radiomics-Based Nomogram Integrating Skeletal Muscle Assessment for Predicting Pathologic Complete Response to Neoadjuvant Therapy in Triple-Negative Breast Cancer
Source: Diagnostics (Basel). 2026 Nov 6;16(21):3679. doi: 10.3390/diagnostics16213679 (PMC13763390; doi:10.3390/diagnostics16213679)
Supplement: Supplementary file 1 [file diagnostics-16-03679-s001.zip › diagnostics-4664296-supplementary proofreading final/Supplementary Methods.pdf]

Supplementary Methods

Supplementary Method S1. Abdominopelvic CT Acquisition.

Abdominopelvic CT examinations were performed using multidetector scanners (SOMATOM Definition Edge and SOMATOM Definition Force, Siemens Healthineers, Erlangen, Germany; Discovery CT750 HD, GE Healthcare, Waukesha, WI, USA). The routine protocol consisted of a single portal venous phase. Patients received approximately 0.521 gI/kg of iodinated contrast medium, including Ioversol (Optiray® 320; Guerbet, Paris, France), Iohexol (Iobrix® 300; Taejoon Pharmaceutics, Seoul, Korea), Iomeprol (Iomeron® 350; Bracco Imaging, Milan, Italy), or Iopromide (Ultravist® 300; Bayer AG, Berlin, Germany), injected at 2–2.5 mL/s via an antecubital vein, followed by a 20 mL saline flush. Scanning began 75 s after injection and extended from the lung bases to 5 cm below the ischial tuberosities. Acquisition parameters included tube voltage 70–100 kVp, tube current–time product 167–708 mAs, pitch 0.7–1.1, rotation time 0.5–0.6 s, and slice thickness 5 mm without overlap. Automatic tube voltage selection and current modulation were applied to all scans.

Supplementary Method S2. Breast MRI Acquisition and Post-processing.

Protocol component	Vida (Siemens)	Common settings / notes
**General MRI setup**	3.0 T<br>Patient position: Prone<br>Breast coil model/type: Breast-18ch<br>Number of channels: 18	Dedicated bilateral breast coil
**T2-weighted imaging**	Sequence: TSE Dixon<br>TR/TE (ms): 5950 / 94<br>FOV (mm): 320 x 320<br>Matrix: 448 x 314<br>Slice thickness (mm): 3	-
**Diffusion-weighted imaging**	Sequence: RESOLVE<br>TR/TE (ms): 6490 / 61<br>FOV (mm): 350 x 210<br>Matrix: 256 x 154<br>In-plane resolution (mm): 1.37 x 1.37<br>Slice thickness (mm): 3<br>Acquisition time: 6 min 11 s<br>b values (s/mm2): 0, 500, 1000	-
**DCE T1-weighted imaging**	Sequence: 3D VIBE<br>TR/TE (ms): 4.68 / 2.27<br>FOV (mm): 340 x 340<br>Matrix: 448 x 358<br>Slice thickness (mm): 1	Fat suppression for all DCE acquisitions
Ultrafast acquisition	Start time after contrast administration: 10 s<br>Temporal resolution: 4.18 s<br>Duration: 1 min 25 s<br>Reconstruction: compressed sensing	Continuous ultrafast series acquired before the five conventional phases.
Conventional dynamic acquisition	Start times after contrast administration (s): 93, 176, 259, 342, and 425<br>Number of phases: 5	Five conventional postcontrast phases followed the ultrafast series.
**Post-processing**	Software: Syngo MR XA31	MIP and subtraction images were generated for each postcontrast phase.
**Contrast agent**	Gadobutrol (Gadovist), 0.1 mmol/kg IV	-
