# Supplementary material for: MRI Radiomics-Based Nomogram Integrating Skeletal Muscle Assessment for Predicting Pathologic Complete Response to Neoadjuvant Therapy in Triple-Negative Breast Cancer
Source: Diagnostics (Basel). 2026 Nov 6;16(21):3679. doi: 10.3390/diagnostics16213679 (PMC13763390; doi:10.3390/diagnostics16213679)
Supplement: Supplementary file 1 [file diagnostics-16-03679-s001.zip › diagnostics-4664296-supplementary proofreading final/Supplementary Table S1-S11 and Figure S1.pdf]

Supplementary Tables

Table S1. Selected radiomic features and corresponding coefficients used for radiomic score construction.

Selected feature	Image transformation	Feature class	Coefficient
log_sigma_4_0_mm_3D_glszm_SizeZoneNonUniformity	Log-sigma	GLSZM	-0.0513
log_sigma_5_0_mm_3D_glszm_GrayLevelNonUniformity	Log-sigma	GLSZM	-0.0674
wavelet_LHL_glszm_GrayLevelNonUniformityNormalized	Wavelet-LHL	GLSZM	0.0564
wavelet_HLL_glcm_Correlation	Wavelet-HLL	GLCM	-0.0143
wavelet_HLL_glcm_ClusterTendency	Wavelet-HLL	GLCM	-0.0896
wavelet_HHH_firstorder_Mean	Wavelet-HHH	First-order	0.112
wavelet_LLL_glszm_SmallAreaLowGrayLevelEmphasis	Wavelet-LLL	GLSZM	-0.0071

Intercept = -0.0463

GLCM = gray-level co-occurrence matrix; GLSZM = gray-level size zone matrix; H = high-pass filter; L = low-pass filter.

Table S2. Multicollinearity diagnostics for integrated models.

A. Variance inflation factors and tolerance.

Model / predictor	VIF	Tolerance
**Model 4**		
Radiomic score	1.03	0.97
NACI vs. NAC	1.05	0.95
Ki-67	1.02	0.98
**Model 6**		
Radiomic score	1.05	0.95
NACI vs. NAC	1.08	0.93
Ki-67	1.03	0.97
SMD	1.07	0.93

VIF = variance inflation factor; NACI = neoadjuvant chemoimmunotherapy; NAC = neoadjuvant chemotherapy; SMD = skeletal muscle density.

B. Spearman rank-correlation matrix.

Variable	Radiomic score	NACI vs. NAC	Ki-67	SMD
Radiomic score	1	0.19 (0.05, 0.33)	0.13 (−0.01, 0.28)	0.22 (0.08, 0.36)
NACI vs. NAC		1	0.12 (−0.03, 0.27)	0.20 (0.06, 0.34)
Ki-67			1	0.10 (−0.05, 0.24)
SMD				1

Values are Spearman correlation coefficients (95% CIs). For this correlation analysis, treatment was coded as NACI = 1 and NAC = 0.

Table S3. Incremental predictive value of SMD.

A. Model performance.

Model	AUC (95% CI)	Hosmer–Lemeshow p	AIC	Brier score (95% CI)
Model 1: radiomic score	0.728 (0.655–0.802)	0.104	209.533	0.209 (0.184–0.234)
Model 5: radiomic score + SMD	0.747 (0.675–0.819)	0.557	199.509	0.203 (0.176–0.229)
Model 4: radiomic score + treatment + Ki-67	0.784 (0.717–0.851)	0.979	186.275	0.190 (0.161–0.219)
Model 6: Model 4 + SMD	0.793 (0.727–0.859)	0.557	178.284	0.187 (0.158–0.217)

B. Nested-model comparisons.

Comparison	ΔAUC (95% CI)	p	Likelihood-ratio p	IDI (95% CI)	p	Continuous NRI (95% CI)	p
Model 5 vs. Model 1	0.019 (−0.015 to 0.053)	0.274	< 0.001	0.022 (0.000–0.044)	0.047	0.139 (−0.158 to 0.435)	0.361
Model 6 vs. Model 4	0.009 (−0.013 to 0.030)	0.428	0.002	0.011 (−0.005 to 0.027)	0.165	0.049 (−0.248 to 0.345)	0.748

Confidence intervals for the Brier score were calculated using a normal approximation based on efficient influence functions. Likelihood-ratio p-values were derived from AIC differences between Firth penalized logistic regression models, adjusted for the difference in parameter count. These comparisons are interpreted cautiously and are not considered sufficient evidence of incremental predictive value.

Table S4. Radiomic-feature selection frequencies across 1000 bootstrap resamples.

A. Top 15 radiomic features by selection frequency.

Rank	Radiomic feature	Selection frequency, n (%)
1	wavelet_HHH_firstorder_Mean*	939 (93.9)
2	wavelet_LHL_glszm_GrayLevelNonUniformityNormalized*	873 (87.3)
3	log_sigma_4_0_mm_3D_glszm_SizeZoneNonUniformity*	807 (80.7)
4	wavelet_LHL_glcm_Imc2	738 (73.8)
5	wavelet_LLL_glszm_GrayLevelNonUniformityNormalized	661 (66.1)
6	wavelet_LLL_glszm_SmallAreaHighGrayLevelEmphasis	631 (63.1)
7	log_sigma_4_0_mm_3D_glszm_GrayLevelNonUniformityNormalized	611 (61.1)
8	log_sigma_3_0_mm_3D_firstorder_Kurtosis	604 (60.4)
9	wavelet_HHH_glszm_SizeZoneNonUniformityNormalized	578 (57.8)
10	wavelet_LHL_glrlm_ShortRunLowGrayLevelEmphasis	525 (52.5)
11	wavelet_LLL_firstorder_10Percentile	519 (51.9)
12	wavelet_HHH_glszm_GrayLevelNonUniformityNormalized	486 (48.6)
13	log_sigma_2_0_mm_3D_glrlm_RunVariance	485 (48.5)
14	wavelet_HLH_glszm_HighGrayLevelZoneEmphasis	458 (45.8)
15	wavelet_LHL_glszm_ZoneEntropy	425 (42.5)

B. Radiomic features ranked 16–30 and additional original-score features.

Rank	Radiomic feature	Selection frequency, n (%)
16	log_sigma_3_0_mm_3D_glcm_Imc1	416 (41.6)
17	wavelet_LHH_glszm_HighGrayLevelZoneEmphasis	407 (40.7)
18	wavelet_HLH_firstorder_Mean	390 (39.0)
19	wavelet_LLL_glszm_ZoneVariance	387 (38.7)
20	original_glszm_SmallAreaHighGrayLevelEmphasis	370 (37.0)
21	wavelet_HHL_glszm_SizeZoneNonUniformityNormalized	370 (37.0)
22	wavelet_HHL_firstorder_Range	367 (36.7)
23	wavelet_LLL_glszm_SmallAreaLowGrayLevelEmphasis*	366 (36.6)
24	wavelet_HLL_glszm_GrayLevelNonUniformityNormalized	363 (36.3)
25	wavelet_LHH_glszm_SmallAreaHighGrayLevelEmphasis	356 (35.6)
26	wavelet_HLH_glcm_Autocorrelation	353 (35.3)
27	wavelet_HHL_glszm_GrayLevelNonUniformityNormalized	326 (32.6)
28	wavelet_HLL_glcm_ClusterTendency*	324 (32.4)
29	wavelet_HLH_firstorder_Median	319 (31.9)
30	log_sigma_4_0_mm_3D_glcm_Correlation	316 (31.6)
46	log_sigma_5_0_mm_3D_glszm_GrayLevelNonUniformity*	229 (22.9)
94	wavelet_HLL_glcm_Correlation*	151 (15.1)

Within each bootstrap resample, LASSO was repeated with 10-fold cross-validation using lambda.min; features with nonzero coefficients were counted as selected. *Features included in the original seven-feature radiomic score. Ranks 46 and 94 are shown because these were original-score features outside the top 30.

Table S5. Final-model selection frequencies across 1000 bootstrap resamples.

**Selected final model**	**Selection frequency, n (%)**
Model 1: radiomic-score model	107 (10.7)
Model 2: clinicopathologic model	1 (0.1)
Model 3: skeletal muscle model	0 (0.0)
Model 4: Model 1 + Model 2	302 (30.2)
Model 5: Model 1 + Model 3	87 (8.7)
Model 6: Model 1 + Model 2 + Model 3	503 (50.3)

Within each bootstrap resample, Models 1–6 were reconstructed and the model with the highest AUC was recorded as the selected final model. AUC = area under the receiver operating characteristic curve.

Table S6. Variable inclusion frequencies in selected final models across 1000 bootstrap resamples.

**Variable**	**Selection frequency, n (%)**
Radiomic score*	999 (99.9)
Ki-67*	603 (60.3)
SMD*	558 (55.8)
Treatment*	508 (50.8)
Tumor grade	367 (36.7)
SMA	202 (20.2)
Family history of breast cancer	180 (18.0)
Clinical T category	107 (10.7)
Clinical N category	62 (6.2)
BMI	60 (6.0)
SMI	56 (5.6)
Age	53 (5.3)
Menopausal status	45 (4.5)
Clinical stage	43 (4.3)
BRCA1/2 mutation status	41 (4.1)
Tumor histology	4 (0.4)

The radiomic score was reconstructed within each bootstrap resample using features with nonzero LASSO coefficients. *Variables included in the original final Model 6. BMI = body mass index; SMA = skeletal muscle area; SMD = skeletal muscle density; SMI = skeletal muscle index.

Table S7. Regression coefficients and prediction equation for Model 6.

Term	Coding or unit	β coefficient
Intercept	—	-3.30
Radiomic score	Original continuous radiomic-score scale	4.45
Treatment	NAC = 0; NACI = 1	0.59
Ki-67	Per 1% increase	0.02
SMD	Per 1 HU increase	0.04

Linear predictor = $\beta_0 + \beta_1 \times$ radiomic score $+ \beta_2 \times$ treatment $+ \beta_3 \times$ Ki-67 $+ \beta_4 \times$ SMD.

Predicted probability of pCR = exp(linear predictor)/[1 + exp(linear predictor)].

Coefficients are rounded to two decimal places; predicted probabilities calculated using these coefficients are approximate.

Table S8. Treatment–radiomic score analyses.

A. Radiomic score by treatment group.

Variable	NACI (n = 94)	NAC (n = 80)	p value
Radiomic score	−0.02 (−0.12 to 0.08)	−0.09 (−0.19 to 0.03)	0.010

Data are medians (IQRs). The p value was calculated using the Wilcoxon rank-sum test.

B. Radiomic score according to pCR, stratified by treatment group.

Treatment	Non-pCR	pCR	p value
NACI	−0.08 (−0.19 to 0.01)	0.01 (−0.07 to 0.11)	0.002
NAC	−0.13 (−0.25 to −0.02)	−0.01 (−0.10 to 0.13)	<0.001

NACI: non-pCR n = 38 and pCR n = 56. NAC: non-pCR n = 51 and pCR n = 29. Data are medians (IQRs).

C. Treatment-stratified association and discrimination.

Treatment	OR per SD (95% CI)	p value	AUC (95% CI)
NACI	2.53 (1.39–4.63)	0.003	0.692 (0.583–0.801)
NAC	2.79 (1.45–5.37)	0.002	0.748 (0.640–0.856)

Table S9. Availability and Missingness of PD-L1 Data.

A. Availability of PD-L1 data according to year of surgery.

**Operation year**	**Total**	**PD-L1 available**	**PD-L1 missing**
2021	22	4 (18.2%)	18 (81.8%)
2022	40	25 (62.5%)	15 (37.5%)
2023	52	45 (86.5%)	7 (13.5%)
2024	60	48 (80.0%)	12 (20.0%)
Total	174	122 (70.1%)	52 (29.9%)

B. Comparison of patients with available and missing PD-L1 data.

Variable	PD-L1 available	PD-L1 missing	p value
Number of patients	122	52	
**Treatment**			**<0.001**
NACI	81 (66.4)	13 (25.0)	
NAC	41 (33.6)	39 (75.0)	
**pCR outcome**			**0.642**
Non-pCR	61 (50.0)	28 (53.8)	
pCR	61 (50.0)	24 (46.2)	
**Age**			**0.478**
<40 years	14 (11.5)	8 (15.4)	
≥40 years	108 (88.5)	44 (84.6)	
**Clinical T category**			**0.504**
T1	4 (3.3)	1 (1.9)	
T2	92 (75.4)	39 (75.0)	
T3	24 (19.7)	9 (17.3)	
T4	2 (1.6)	3 (5.8)	
**Clinical N category**			**0.700**
N0	30 (24.6)	16 (30.8)	
N1	52 (42.6)	23 (44.2)	
N2	18 (14.8)	5 (9.6)	
N3	22 (18.0)	8 (15.4)	
**Clinical stage**			**0.204**
II	72 (59.0)	36 (69.2)	
III	50 (41.0)	16 (30.8)	
Ki-67	61.6 (43.0–77.0)	52.0 (34.5–70.5)	0.075
SMD	49.0 (44.0–54.0)	47.0 (42.0–52.0)	0.117
Radiomic score	−0.04 (−0.14 to 0.06)	−0.04 (−0.18 to 0.07)	0.993

Data are n (%) or median (IQR). P values were calculated using chi-square, Fisher exact, or Wilcoxon rank-sum tests, as appropriate.

Table S10. Complete-case models incorporating PD-L1 (n = 122).

A. Multivariable logistic regression.

Model / predictor	β coefficient ± SE	Adjusted OR (95% CI)	p value
**Model 6**			
Radiomic score	3.68 ± 1.28	2.03 (1.25–3.28)	0.004
NACI vs. NAC	0.91 ± 0.46	2.48 (1.01–6.08)	0.048
Ki-67	0.04 ± 0.01	1.04 (1.02–1.06)	0.001
SMD	0.03 ± 0.03	1.03 (0.97–1.09)	0.337
**Model 7-1: Model 6 + continuous PD-L1 CPS**			
Radiomic score	3.20 ± 1.26	1.85 (1.15–2.97)	0.011
NACI vs. NAC	0.82 ± 0.46	2.27 (0.92–5.63)	0.076
Ki-67	0.04 ± 0.01	1.04 (1.01–1.06)	0.001
SMD	0.04 ± 0.03	1.04 (0.98–1.10)	0.223
PD-L1 CPS, continuous	0.02 ± 0.01	1.02 (1.00–1.05)	0.084
**Model 7-2: Model 6 + PD-L1 CPS ≥ 10**			
Radiomic score	3.29 ± 1.29	1.88 (1.16–3.05)	0.011
NACI vs. NAC	0.85 ± 0.46	2.34 (0.94–5.80)	0.066
Ki-67	0.04 ± 0.01	1.04 (1.01–1.06)	0.001
SMD	0.03 ± 0.03	1.03 (0.97–1.09)	0.321
PD-L1 CPS ≥ 10	0.57 ± 0.47	1.76 (0.70–4.44)	0.230

Radiomic-score ORs are reported per one-SD increase; Ki-67, SMD, and continuous PD-L1 CPS ORs are per one-unit increase. NAC is the treatment reference.

B. Model performance.

**Model**	**AUC (95% CI)**	**Hosmer–Lemeshow p**	**ΔAUC vs. Model 6**	**p value**
Model 6	0.790 (0.712–0.868)	0.230	Reference	Reference
Model 7-1	0.806 (0.730–0.882)	0.683	0.016 (−0.017 to 0.049)	0.343
Model 7-2	0.797 (0.720–0.874)	0.567	0.007 (−0.014 to 0.028)	0.498

Table S11. NACI subgroup analyses among patients with available PD-L1 data (n = 81).

A. Predictor distributions according to pCR.

**Predictor**	**Non-pCR (n = 34)**	**pCR (n = 47)**	**p value**
Radiomic score	−0.08 (−0.19 to 0.01)	0.00 (−0.09 to 0.10)	0.018
Ki-67	53.0 (39.0–67.0)	70.0 (51.0–80.0)	0.006
SMD	48.0 (43.0–53.0)	51.0 (46.0–54.0)	0.071
PD-L1 CPS	10.0 (0.0–10.0)	15.0 (10.0–30.0)	0.009
**PD-L1 CPS category**			**0.102**
<10	12 (35.3)	9 (19.1)	
≥10	22 (64.7)	38 (80.9)	

B. Logistic regression.

**Predictor**	**Univariable OR**	**p**	**Adjusted OR: continuous CPS**	**p**	**Adjusted OR: CPS ≥ 10**	**p**
Radiomic score	2.11 (1.15–3.88)	0.016	1.89 (1.05–3.39)	0.033	1.96 (1.08–3.58)	0.027
Ki-67	1.03 (1.01–1.05)	0.013	1.03 (1.01–1.06)	0.013	1.04 (1.01–1.06)	0.009
SMD	1.07 (1.00–1.14)	0.047	1.07 (1.00–1.16)	0.065	1.06 (0.99–1.14)	0.113
PD-L1 CPS, continuous	1.03 (1.00–1.05)	0.068	1.02 (0.99–1.05)	0.147		
PD-L1 CPS ≥ 10	2.25 (0.82–6.18)	0.115			1.60 (0.51–4.97)	0.418

Radiomic-score ORs are per one-SD increase; other continuous-predictor ORs are per one-unit increase.

Supplementary Figure

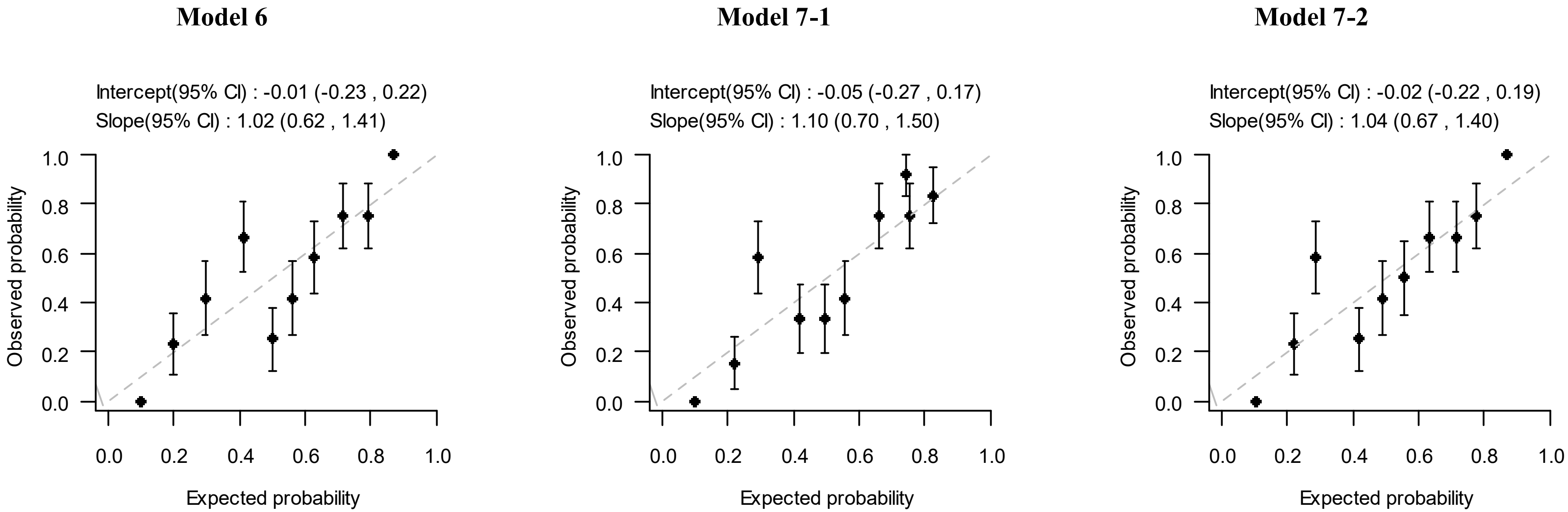

Figure S1. Calibration plots for complete-case models.
Apparent calibration plots for complete-case models in the 122 patients with available PD-L1 data. Model 6 includes the radiomic score, treatment type, Ki-67, and SMD. Model 7-1 additionally includes continuous PD-L1 CPS, whereas Model 7-2 additionally includes categorical PD-L1 CPS (≥ 10 vs. < 10). Dashed diagonal lines represent perfect calibration.
